# Osteopontin Regulates Endometrial Stromal Cell Migration in Endometriosis through the PI3K Pathway: Osteopontin Regulates Endometrial Cell Migration in Endometriosis

**DOI:** 10.1007/s43032-020-00301-8

**Published:** 2020-09-09

**Authors:** Xiaoxia Fu, Mengyun Yao, Chaoshuang Ye, Tao Fang, Ruijin Wu

**Affiliations:** 1 Department of Obstetrics and Gynecology, Women’s Hospital, Zhejiang University School of Medicine, Hangzhou, 310006 Zhejiang Province People’s Republic of China; 2 Institute of Burn Research, South-West Hospital, State Key Lab of Trauma, Burn and Combined Injury, Chongqing Key Laboratory for Disease Proteomics, Third Military Medical University, Chongqing, China

**Keywords:** Osteopontin, endometriosis, cell migration, PI3K, uPA

## Abstract

Endometriosis is generally characterized as a tumor-like disease because of its potential for distant metastasis and local tissue invasion, while whether osteopontin (OPN) plays a role in the pathogenesis of endometriosis has not been thoroughly investigated. We investigated the expression of OPN, urokinase plasminogen activator (uPA), phosphatidylinositol 3 kinase (PI3K), and phospho-PI3 kinase (p-PI3K) in endometrial stromal cells (ESCs). The serum concentration of OPN was determined by enzyme-linked immunosorbent assays (ELISA). OPN was downregulated to explore the corresponding change of uPA, p-PI3K, F-actin, and α-tubulin. The expression of OPN, uPA, PI3K, and p-PI3K was evaluated by western blot and quantitative real-time PCR (RT-qPCR) and the expression of F-actin and α-tubulin was confirmed by immunofluorescence assay. The proliferation and migration abilities of ESCs were investigated by CCK8, transwell, and wound scratch assays. Endometrial OPN, p-PI3K, and uPA expressions and serum OPN levels were increased in patients with endometriosis compared with the control. The expressions of p-PI3K, uPA, and α-tubulin were decreased by siRNA-OPN interference in ectopic ESCs. Activation and inhibition of the PI3K pathway apparently upregulate and downregulate uPA expression. Knockdown of OPN and inhibition of the PI3K pathway remarkably inhibited cell migration in ectopic ESCs. Meanwhile, activation of the PI3K pathway promoted the migration ability of ectopic ESCs. OPN may regulate the expression of uPA through the PI3K signal pathway to affect the migration ability of ESCs, indicating that OPN, uPA, and the PI3K pathway may be potential targets for interrupting development of endometriosis.

## Introduction

Endometriosis, defined as the implantation and periodic growth of endometrial glands and stroma at extra-uterine sites, is a common benign gynecological disease with a heavy social and economic burden since symptoms of endometriosis include chronic pelvic pain, dysmenorrhea, dyspareunia, and infertility. Retrograde menstruation is the most widely accepted theory put forward to explain the pathogenesis of endometriosis [1]. However, retrograde menstruation is observed in most women of reproductive age [2], while endometriosis occurs in only 8–10% [3]. This fact suggests that other factors are involved in the establishment of endometriosis. To this day, endometriosis is considered a multifactorial disease with undetermined etiology. Complex interaction of genetic, immunologic, endocrine, and environmental factors is matter to the susceptibility to endometriosis.

Endometriosis is characterized as a benign disease with some properties of malignant tissues such as hyperplasia, cell invasion, and induction of metastasis [4–9]. This fact suggests that certain signaling molecules and corresponding signaling pathways, playing roles in invasion and metastasis of many commonly occurring cancers, might be also involved in endometriosis.

Osteopontin (OPN), also called secreted phosphoprotein 1 (SPP-1), is a 70-kDa secreted phosphorylated glycoprotein which is one of the important molecular targets in cancer progression and metastasis [10]. It was originally isolated from bone matrix [11] and has been found in a variety of tissues including kidney, brain, pancreas, lung bronchi, secretory glands, salivary glands, lactating breast, and some tumor tissues [12–16]. It is also secreted by activated immunocytes such as macrophages, lymphocytes, T lymphocytes, natural killer cells, and Kupffer cells [17]. OPN contains binding sites for αvβ3 integrin, thrombin, glycosylation, calciumions, and heparin [12]. Previous studies have shown that OPN is over expressed in a wide range of malignant tumors such as lung cancer [18], endometrial carcinoma [19], breast cancer [20, 21], and melanoma [22], implicated in anti-apoptosis, angiogenesis, cell adhesion, and migration [19, 23]. It was reported that OPN mediated the progression of ovarian cancer through the activation of the PI3K/Akt (protein kinase B) pathway [24] and promoted hepatocellular carcinoma and mammary cancer invasion by upregulating the expression of uPA [25, 26]. Another research found that OPN leads to uPA activation and promoted proliferation, apoptosis, invasion, and migration in gastric cancer, regulated by the PI3K pathway [27]. The OPN/PI3K/uPA pathway represents a new potential molecular mechanism that might be involved in cancer progression.

Recently, OPN was reported to be related with endometriosis [28–31]. A complementary deoxyribonucleic acid (cDNA) microarray analysis showed that the transcription of OPN in ectopic endometrial lesions was 130-fold higher than that in normal endometrial samples [32]. It was further certified that OPN enhances endometrial cell invasion and contribute to the establishment of ectopic foci [28]. However, the roles for OPN and PI3K signaling pathway and uPA in the pathogenesis of endometriosis still remain largely unexplored. The present study aimed to explore the effect of OPN and related proteins on migration abilities of endometrial stromal cells (ESCs) from patients with endometriosis. The presence of relevant protein and their gene expression in specimens was verified by western blotting and RT-qPCR. Transwell, wound scratch, and cell counting kit-8 (CCK8) assays were conducted to evaluate the migration and proliferation abilities of ESCs.

## Materials and Methods

### Participants’ Recruitment and Specimens

Ethics approval was granted by the Ethics Committee of Women’s Hospital School of Medicine Zhejiang University (reference number 20130039) on June 2013 and all participants signed a written informed consent prior to inclusion. For the purpose of this study, fresh endometriosis cyst tissues and serum were collected from participants enrolled in the hospital from Jan 2016 to Dec 2018. Totally 68 women (aged 22–50 years, average 34.1 years) with III/IV stage endometriosis (48 in stage III and 20 in stage IV) and 18 women (study group, aged 24–49 years, average 31.4 years) without visible endometriosis (control group) undergoing operation for hydrosalpinx, septate uterus, endometrial polyp anastomosis of tube, or other benign gynecological diseases were recruited for this study. There is no significant difference in body mass index, parity, and progesterone level between two groups. Patients with adenomyosis, uterine fibroids, hypertension, diabetes mellitus, cardiopathy, other neoplastic, and serious diseases were excluded. All participants had regular menstrual cycles between 25 and 35 days in duration and none of them had been prescribed hormones or medications known to influence reproductive functions for at least 6 months before the surgery. About 3 ml venous serum was drawn before surgery and the endometrial tissues were transferred to the sterile phosphate buffer saline (PBS) and transported to the laboratory on ice in an hour for further experiments.

### Enzyme-Linked Immunosorbent Assay

Peripheral blood samples were drawn from the 68 patients before surgical operation and only 46 blood samples (serum) were redundant enough for our following enzyme-linked immunosorbent assay (ELISA) experiment after clinical regular detect.

The concentration of OPN in the serum from 46/68 patients with endometriosis and 18 control patients was measured in accordance with the instruction of the ELISA kit (R&D Systems, No.DOST00, Minneapolis, USA). The optical density was detected by a microplate reader at 450 nm and the serum concentration of OPN would be computed by referring the standard curve.

### Primary Culture of Endometrial Stromal Cells

Our rationale for using in vitro cultured ESCs as a model for endometriosis research have been generally accepted [33–36]. Briefly, sixty-eight ectopic endometrial cysts from 68 women with III/IV stage endometriosis were collected, among them we also collected 12 homologous eutopic endometria. Nine endometrial tissues from 18 patients without endometriosis (control endometrium) at proliferation phase were collected from participants by laparoscopy and hysteroscopy. In order to avoid ovarian contamination of ectopic tissue, lining of chocolate cyst was scraped and the fibrous capsule was discarded. After being washed in sterile PBS for three times, the specimens were dissected into small pieces and digested in PBS with collagenase IV (0.05%; Sigma, St. Louis, MO, USA) and deoxyribonuclease I (DNase I) (10 U/mL, Sigma, St. Louis, MO, USA) for 1 h. Residual tissue pieces were removed by being centrifuged for 5 min and 100- and 40-μm cell filters were used to eliminate cell debris and epithelial cells respectively. Isolated cells were suspended in Dulbecco’s Modified Eagle Medium: Nutrient Mixture F-12 (DMEM/F12), supplemented with 10% fetal bovine serum (FBS) and cultured in a cell incubator at 37 °C. ESCs from different patients were kept separate, and each specimen represents an independent individual. Purity of both ectopic and eutopic stromal cells from 5 endometriosis patients were examined by immunocytochemistry staining. Pan cytokeratin (ab7753, Abcam, Cambridge, UK) and vimentin (ab92547, Abcam, Cambridge, UK) were chosen as epithelial and stromal marker, respectively. Both primary cultured EuESC and EcESC, of which the purity were guaranteed to be over 95%, were selected for next steps of experiments. All used ESCs were controlled within passage 4, with the maximum culture time of 15 days.

### Gene Silencing

ESCs were divided into three groups (siRNA-OPN, siRNA scrambled, and control group). Scrambled siRNA was used to set a negative control. The siRNA sequence of OPN designed as follows: sense strand, 5 ′-CUCCAGAGGAUGUUCAAUATT-3′, and antisense strand, 5 ′-UAUUGAACAUCCUCUGGAGTT-3′. The siRNA scrambled is a disordered small RNA. Milli Q water was added to the control group instead of siRNA. All of the siRNAs were designed and purchased from Shanghai Gene-Pharma Inc. RNAiMAX Transfection Reagent (Thermo Fisher Scientific, USA) was used to transfect these siRNA oligonucleotides into ESCs. The ratio of transfection reagent to siRNA was 10 μl:2.5 μg per 2 × 10^5^ cells. For transfection procedure, we followed the protocol provided by the transfection reagent manufacturer. Subsequent western blot was used to evaluate the transfection efficiency after 48 h.

### RNA Extraction and RT-qPCR

Total RNA was extracted from the ESCs using TRIzol reagent (Invitrogen, USA), chloroform, isopropanol, and diethyl pyrocarbonate (DEPC)–treated water according to the manufacturer’s instruction. A NanoDrop spectrophotometer (Thermo Scientific, Waltham, MA, USA) was used to detect the RNA concentration. The reverse transcription was accomplished by using PrimeScript™ RT reagent Kit (Takara Bio, Japan) and 1 μg of total RNA was reverse transcribed into cDNA in a 20 μl volume. RT-qPCR was then carried by SYBR Premix Ex Taq™ Kit (Takara Bio, Japan) as follows: 95 °C for 15 s followed by 40 cycles at 95 °C for 5 s and 60 °C for 30 s (for up to 40 cycles). GAPDH was chosen to provide a normalization control. The primers used in above-mentioned experiment are presented in Table 1. For each sample, the average threshold (Ct) was calculated from triplicate wells and the fold change was analyzed using the 2^-ΔΔCt^ method. At least four independent experiments were performed for each condition.

**Table 1:** The primer sequences for OPN, uPA, and GAPDH

Gene	Sequences
OPN forward primer	5′-ACCCTGATGCTACAGACGAG-3′
OPN reverse primer	5′-GACTATCAATCACATCGGAATG-3′
uPA forward primer	5′-CTGTGAGATCACTGGCTTTG-3′
uPA reverse primer	5′-TTGGAGGGAACAGACGAG-3′
GAPDH forward primer	5′-TCAGTGGTGGACCTGAC-3′
GAPDH reverse primer	5′-TGCTGTAGCCAAATTCGTT-3′

### Western Blot

Western blot was accomplished using lysates from ESCs pretreated in different conditions. A total of 1 × 10^7^ ESCs was washed twice with cold PBS and then submerged in 150 μl RIPA Lysis and Extraction Buffer (Thermo Scientific, Waltham, MA, USA) with 1.5 μl phenylmethanesulfonyl fluoride (PMSF) (Thermo Fisher Scientific, USA). The cell lysates were centrifuged at 12,000*g* for 15 min at 4 °C. Bradford method was used to determine the protein concentrations. Equal amounts of lysate (50 μg) were run on 10% sodium dodecyl sulfonate (SDS)-polyacrylamide gels and then transferred to a PVDF transfer membrane (Thermo Scientific, Waltham, MA, USA). The membranes were washed in Tris-buffered saline tween (TBST), blocked in 5% non-fat dry milk in TBST, and incubated with primary OPN (1:1000, Abcam 8448, UK), uPA (1:1000, Abcam 169754, UK), p-PI3K (1:1000, Abcam 182651, UK), and PI3K (1:1000, Abcam 86714, UK) antibodies overnight at 4 °C. GAPDH (1:1000, DawenBiological, China) antibody was used as internal control for normalization. The membranes were then washed three times with TBST and incubated with secondary antibodies (1:5000, Dawen Biological, China) conjugated to horseradish peroxidase for an hour at room temperature. Bands of immune reactive proteins were visualized by the enhanced chemiluminescence (ECL) reagent (BI, Israel) and normalized to the loading internal control. At least four independent experiments were performed for each circumstance.

### Immunofluorescence Assay

ESCs were seeded on creep plates (1 × 10^5^cells/well), fixed with 4% paraformaldehyde for 15 min, permeabilized with 0.1% Triton X-100, and blocked with 1% BSA for 1 h. ESCs were than triple stained with fluoresce in isothiocyanate (FITC)-phalloidin (1:100, Sigma-Aldrich, St. Louis, MO, USA) for 40 min, 4′,6-diamidino-2-2-phenylindole (DAPI) (ab104139, Abcam, Cambridge, UK) for 10 min, and Alexa Fluor 594-anti-alpha tubulin antibody (DM1A, Abcam, Cambridge, UK) at 4 °C overnight. Immunofluorescence photographs were taken with a laser scanning confocal microscope (LSM 510, Zeiss, Oberkochen, Germany) to calculate the pixel (Px) of cells by ImageJ. At least 200 cells from triplicate cover slides in each sample were analyzed and experiments were repeated in samples from four individuals.

### Cell Migration Assay and Effects of Inhibitor and Activator

The ESC migration assay was carried out by transwell and wound scratch assays. A total of 1.5 × 10^5^ ESCs per well were seeded into the upper chamber using FBS-free DMEM/F12. DMEM/F12 medium supplemented with 10% serum was added to the lower chamber. ESCs were also co-cultured with p-PI3K inhibitor ly294002 (50 μM) and p-PI3K activator-SF1670(4 μM) or siRNA-OPN respectively to evaluate the regulation of cell migration. Twenty-four hours later, the upper chamber was removed, washed with PBS, immersed in alcohol for 15 min, and then stained with 0.1% crystal violet solution. Cells at the inner face of the upper chamber were removed with a cotton swab. Cells outside the upper chamber were photographed and counted under a light microscope (200 ×). Cells in five visual fields per chamber were counted and all transwell assays were performed in triplicate for eight independent experiments.

As for wound scratch assays, ESCs were evenly seeded in a 6-well plate (5 × 10^5^ cells/well) and incubated at 37 °C in a 5% CO_2_ incubator. When cell confluence reached 90%, we replaced the medium (DMEM/F12 + 10% FBS) with FBS-free DMEM/F12. Then a ruler and a 10-μl pipette tip were used to generate a straight wound in the 6-well plates [37–39]. Cellular debris was removed by washing the scratched area with PBS for three times. Remaining ESCs were cultured in DMEM/F12 and photographed at 0 h, 24 h, and 48 h under a microscope. At least 5 random areas of the plate were photographed (100 ×) and the migration rates were computed based on the change of wound width measured by ImageJ (NIH, Bethesda, MD). All wound scratch assays were repeated in samples from at least eight different individuals.

### Cell Counting Kit-8 Assay

Treated ESCs were seeded in a 96-well plate (2 × 10^4^ cells/well) and incubated in FBS-DMEM/F12. Cell counting kit-8 (CCK8) reagent (10 μl/well, DOJINDO, CK04-13) was added at 0 h, 24 h, and 48 h. After 4 h of incubation, the optical density (OD) was measured by ultraviolet spectrophotometry at 440 nm. At least seven independent experiments were performed using five wells for each condition.

### Statistics

Data from at least four independent experiments were analyzed using GraphPad Prism 5 (GraphPad Software, San Diego, CA, USA) and presented as mean ± SEM. Sample size calculation was according to formula: *n* = ((*u*_*α*_ + *u*_β_)^2^ × *σ*^2^/*δ*^2^ × (1 + 1/*k*), when *α* = 0.05, *β* = 0.2, *u*_*α*_ = 1.96, *u*_β_ = 0.84, *σ* represents the pooled standard deviation, δ represents the standardized mean difference, the number of one group is *n*, while another group *k* × *n*. A parametric Student *t* test was used for statistical analysis. Multiple comparisons were first analyzed by one-way ANOVA and then by Tukey’s tests. The correlation analyze was used to state the OPN relation between serum and endometrial stromal cells. Values were considered statistically significant if *P* < 0.05 (*P < 0.05, **P < 0.01).

## Results

### Serum Concentrations of OPN

**Fig. 1:**
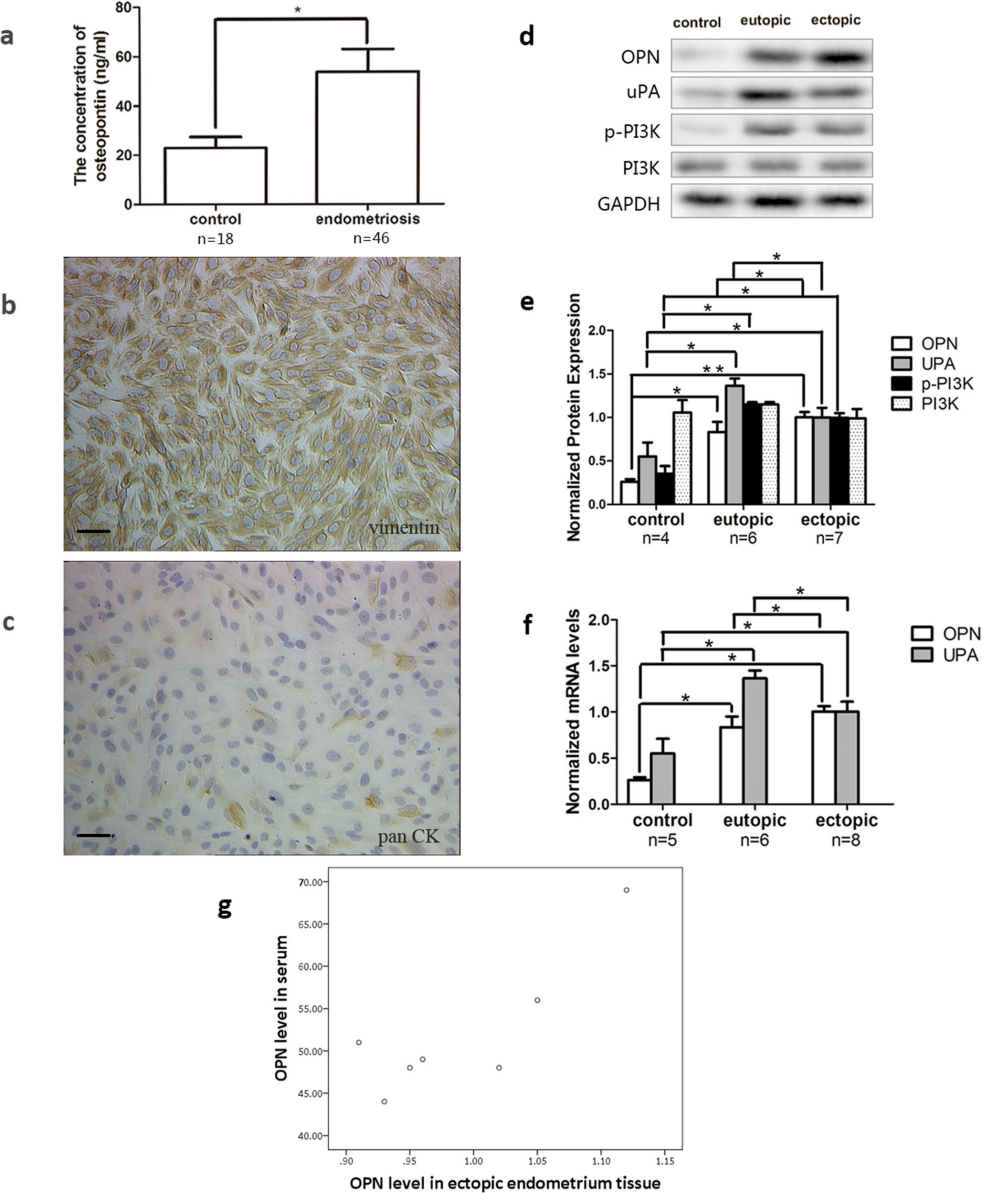
Identification of primary cultured ESCs and expression of OPN, uPA, and PI3K. **a** The serum OPN level was detected by ELISA (control *n* = 18; endometriosis *n* = 46). **b**, **c** Primary cultured ESCs were identified by immunohistochemical staining in EcESC (200 × scale, bar = 100 μm). **d**, **e** The protein (control *n* = 4; eutopic *n* = 6; ectopic *n* = 7) expression of OPN, uPA, PI3K, and p-PI3K in eutopic ESCs (EuESCs), ectopic ESCs (EcESCs), and control ESCs (CoESCs) were examined by western blot. **f** The mRNA (control *n* = 5; eutopic *n* = 6; ectopic *n* = 8) expression of OPN and uPA in EuESCs, EcESCs, and CoESCs were examined by RT-qPCR. **g** The correlation analysis of OPN level between in ectopic endometrium tissue and in serum was displayed (*n* = 7). Results are presented as the mean ± SEM (*p < 0.05, **p < 0.01)

The serum concentration of OPN in patients with endometriosis and the control was detected by ELISA. The average concentration of OPN was 53.83 ± 9.32 ng/ml in the endometriosis group and 22.90 ± 4.7 ng/ml in the control group (Fig. 1a). Statistical analysis showed a significant difference between two groups (*P* < 0.05). There was no significant difference between stage III and IV endometriosis in ELISA results (data unshown).

### Expression of OPN, uPA, PI3K, and p-PI3K in ESCs

Primary ESCs were successfully isolated from endometrial tissues and cultured in dishes. ESCs grew exuberantly and displayed atypical elongated spindle morphology (Fig. 1b, c). Immunocytochemistry was used to identify that the cultured cells were actually ESCs. Randomly selected fields were pictured to calculate the purity. As shown, most cells (over 95%) were positive for vimentin and negative for pan cytokeratin, which means the success of primary culture of ESCs.

Quantification of the OPN, uPA, PI3K, and p-PI3K expression levels in control ESCs (CoESCs), eutopic ESCs (EuESCs), and ectopic ESCs (EcESCs) was performed by RT-qPCR and western blot. GAPDH was chosen as the internal control and the 2^-ΔΔCt^ method was used to normalize mRNA expression. The OPN and uPA mRNA expressions were significantly upregulated in both EuESCs and EcESCs compared with in CoESCs (*P* < 0.05, Fig. 1f). Analogously, we used ratio of the gray density of OPN, uPA, PI3K, and p-PI3K to the gray density of GAPDH to represent the relative protein expression. The protein levels of OPN, uPA, and p-PI3K were significantly higher in both EuESCs and EcESCs than in CoESCs (*P* < 0.05, Fig. 1d, e). However, no significant difference was detected in PI3K protein expression in EuESCs, EcESCs, and CoESCs (*P* > 0.05, Fig. 1d, e). The protein levels of uPA were significantly higher in EuESCs than in EcESCs (*P* < 0.05, Fig. 1d, e). However, OPN was significantly higher in EcESCs than in EuESCs (*P* < 0.05, Fig. 1d, e). No significant difference was detected in p-PI3K and PI3K between EcESCs and EuESCs.

Correlation analyze of OPN level between ectopic endometrium tissue and serum was completed and displayed with a scatter plot (*P* < 0.05, Fig, 1g). The pearson product-moment correlation coefficient is 0.841. There is a positive correlation between tissue OPN and serum OPN.

### Regulation of OPN and PI3K Pathway on Protein Expression in ESCs

**Fig. 2:**
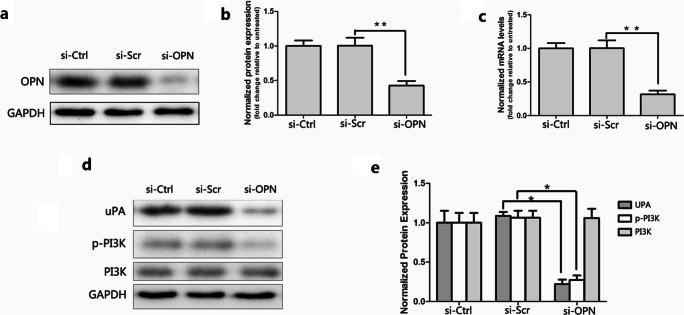
Knockdown of osteopontin (OPN) downregulate the expression of uPA and p-PI3K in EcESCs (si-Ctrl represents control; si-Scr represents siRNA scrambled; si-OPN represents siRNA-OPN; *n* = 4). **a**, **b**, **c** The protein (**a** and **b**) and mRNA (**c**) levels of OPN were detected by western blot and RT-qPCR at 48 h after transfection. **d**, **e** Protein expression of uPA, PI3K, and p-PI3K in untreated EcESCs, siRNA-scrambled treated EcESCs, and siRNA-OPN-treated EcESCs were examined by western blot. Results are presented as the mean ± SEM (*p < 0.05, **p < 0.01)

Knockdown of OPN by siRNA was used to observed uPA and p-PI3K expression change in EcESCs. It was observed that uPA and p-PI3K were regulated by OPN in gastric cancer [27]. To explore the signaling pathways downstream of OPN, we used siRNA to downregulate OPN. OPN-specific siRNA-mediated knockdown of OPN in EcESCs significantly reduced the mRNA and protein levels of OPN at 48-h post transfection (*P* < 0.01, Fig. 2a, b, c). As shown, knockdown of OPN significantly decreased the protein expression of uPA and p-PI3K, while had no effect on PI3K (*P* < 0.05, Fig. 2d, e).

**Fig. 3:**
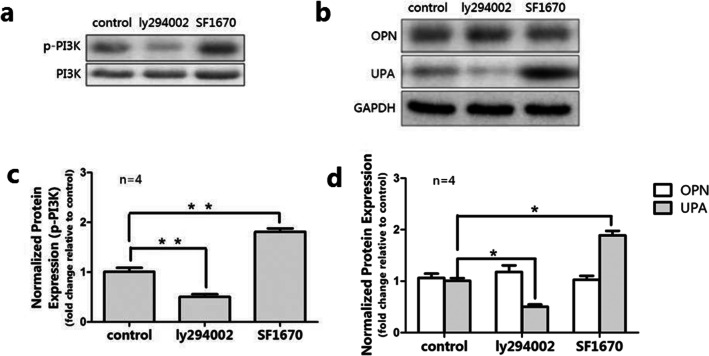
Activation and inhibition of p-PI3K significantly increased and decreased uPA protein expression respectively (*n* = 4). **a**, **c** The protein level of p-PI3K was detected by western blot after 24-h treatment. **b**, **d** Protein expression of uPA and OPN in untreated EcESCs, ly294002-treated EcESCs, and SF1670-treated EcESCs was examined by western blot. Results are presented as the mean ± SEM (*p < 0.05, **p < 0.01)

The PI3K signaling pathway was reported to be involved in the progression of ovarian cancer mediated by OPN [40]. Combining the above-mentioned results, we sought to further explore the role of p-PI3K in EcESCs. The protein expression of p-PI3K was inhibited and activated by ly294002 (50 μM) or SF1670(4 μM) respectively after 12-h (data not shown) and 24-h treatment (Fig. 3a) and statistical analysis based on the gray intensity showed a significant difference (*P* < 0.01, Fig. 3c). As shown, ly294002- and SF1670-mediated downregulated/upregulation of p-PI3K significantly decreased/increased uPA protein expression respectively, meanwhile, showed no effect on OPN protein expression (*P* < 0.05, Fig. 3b, d).

### Effect of OPN on Cytomorphology and Cytoskeleton

**Fig. 4:**
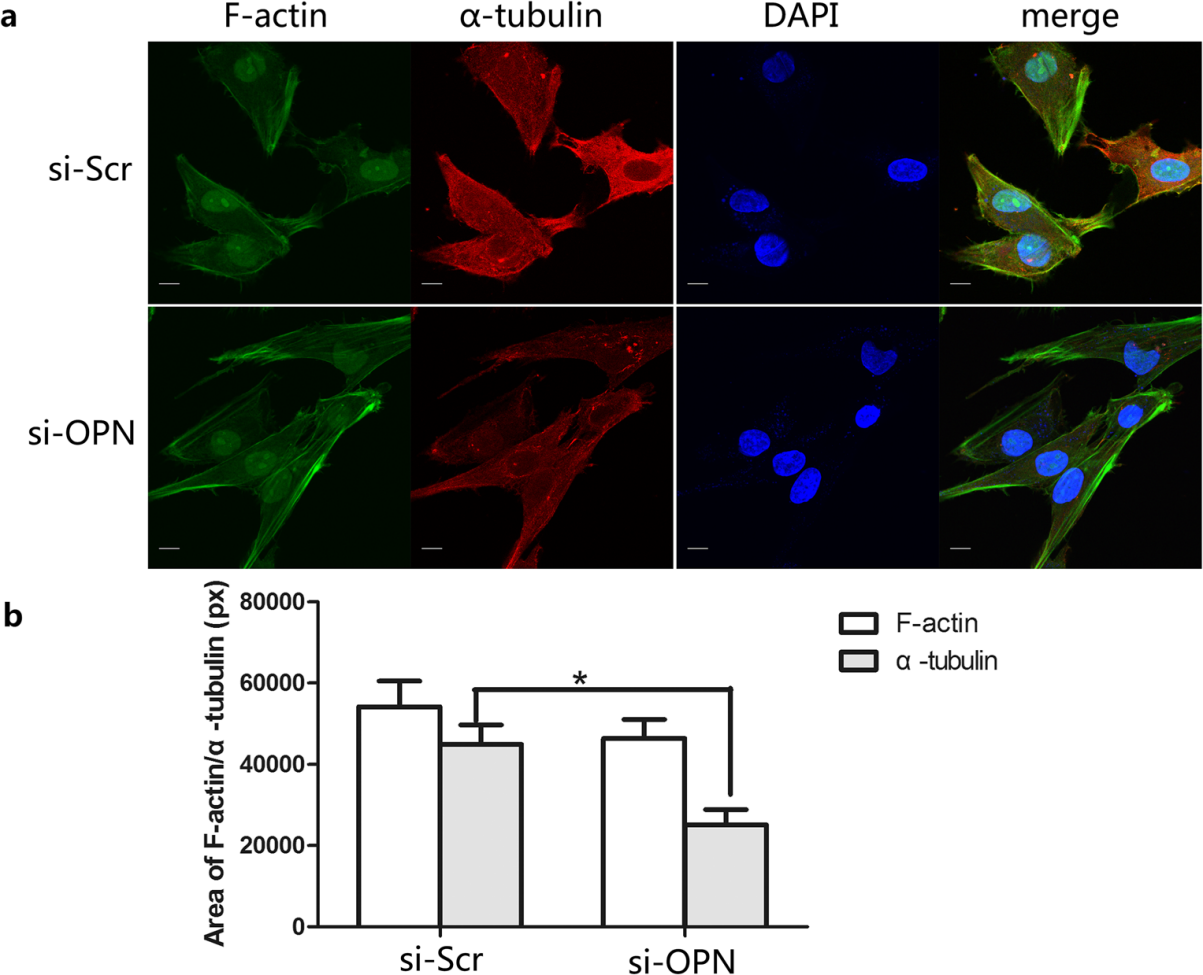
Knockdown of osteopontin (OPN) affected cytomorphology and induced α-tubulin reorganization in EcESCs (si-Scr represents siRNA scrambled; si-OPN represents siRNA-OPN). **a** EcESCs were triple stained with DAPI (for nucleus, blue), FITC-phalloidin (for F-actin, green), and Alexa Fluor 594-DM1A (for α-tubulin, red) at 48 h after siRNA-OPN transfection(1000 × scale, bar = 10 μm, *n* = 4). **b** Color pixels (px represents pixels) of F-actin and α-tubulin were quantified based on more than 200 cells for each experiment and four independent experiments were performed. Results are presented as the mean ± SEM (*p < 0.05)

It was reported that OPN was involved in cytoskeleton dynamics in osteoblast [41] and primary erythroblasts cells [40]. To further explore the role of OPN in cytoskeleton, we knockdown OPN by siRNA and detected subsequent expression of F-actin and α-tubulin by immunofluorescence. EcESCs were fixed with 4% paraformaldehyde and triple stained with DAPI (for nucleus, blue), FITC-phalloidin (for F-actin, green), and Alexa Fluor 594-DM1A (for α-tubulin, red) at 48 h after siRNA-OPN transfection. Decreased lamellipodia and fluorescence intensity of α-tubulin were observed in siRNA-OPN-treated EcESCs (Fig. 4a). Cells treated with siRNA-OPN appears to be a spindle and had more thin edges and corners. The fluorescence intensity of F-actin and α-tubulin was quantified and a significant difference in α-tubulin fluorescence intensity was detected in siRNA-OPN-treated EcESCs compared with siRNA-scrambled treated EcESCs (*P* < 0.05, Fig. 4b). However, no significant difference was observed in F-actin (*P* > 0.05, Fig. 4b).

### Effects of OPN and p-PI3K on EcESCs Migration and Proliferation

**Fig. 5:**
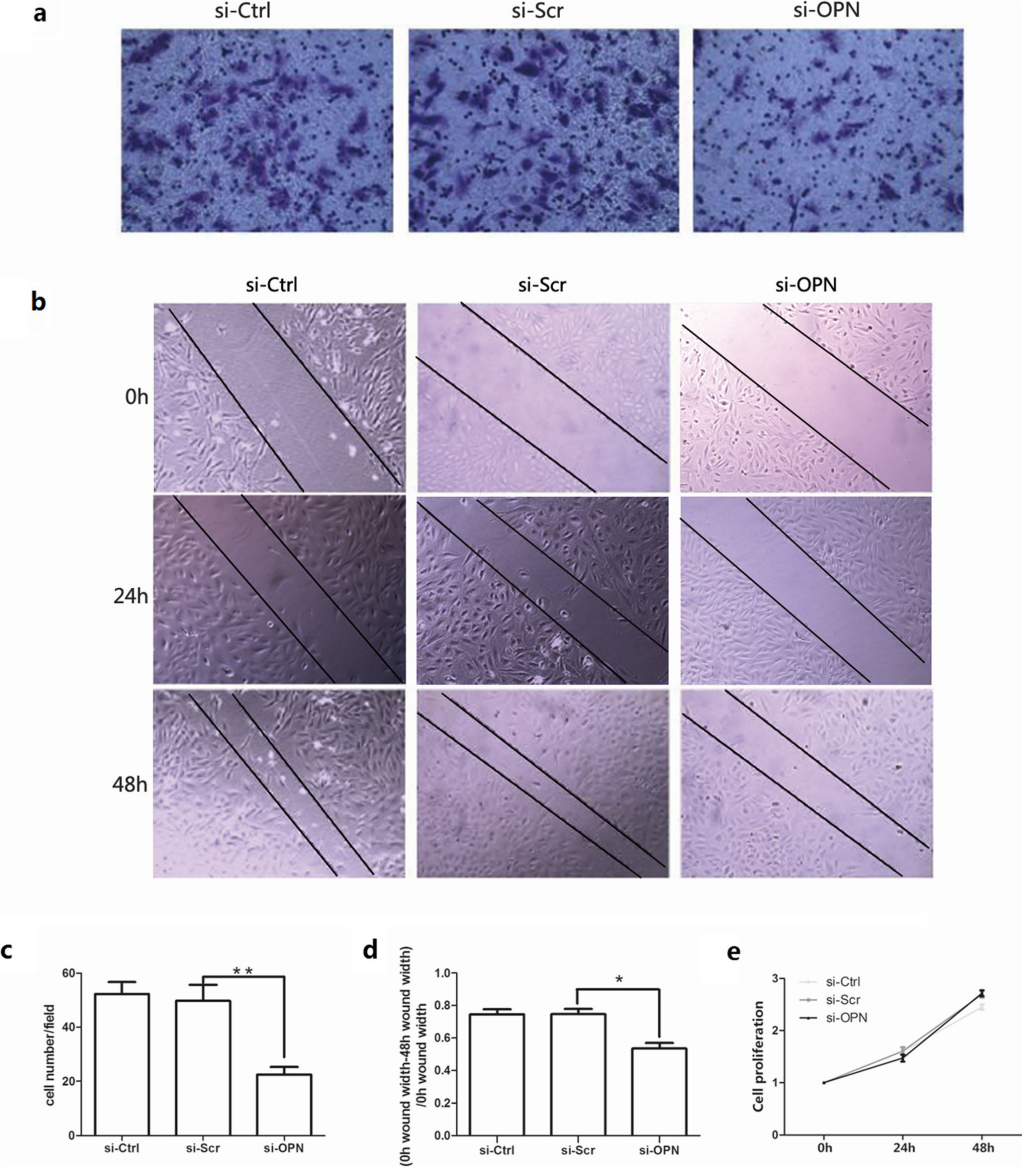
Knockdown of osteopontin (OPN) attenuated cell migration while exhibited no effect on cell proliferation in EcESCs (si-Ctrl represents control; si-Scr represents siRNA scrambled; si-OPN represents siRNA-OPN). **a**, **c** Cell migration was detected by transwell assay (200 × scale, *n* = 8). **b**, **d** Cell migration was detected by wound scratch assay (100 × scale, *n* = 10). **e** Cell proliferation was examined by CCK8 assay (*n* = 7) at 0 h, 24 h, and 48 h after transfection. Results are presented as the mean ± SEM (*p < 0.05, **p < 0.01)

To explore the function of OPN in regulating EcESCs migration and proliferation, we performed transwell, wound scratch, and CCK8 assays. The number of EcESCs outside the upper chamber significantly decreased in the siRNA-OPN group than in the siRNA-scrambled group (*P* < 0.05, Fig. 5a, c). The wound width at 48 h significantly broadened in the siRNA-OPN group than in the siRNA-scrambled group (*P* < 0.05, Fig. 5b, d). Migration ability of EcESCs after OPN downregulation was significantly attenuated in the transwell and the scratch assay, while knockdown of OPN showed no influence on cellular proliferation at all set time points in CCK8 assay (*P* > 0.05, Fig. 3e).

**Fig. 6:**
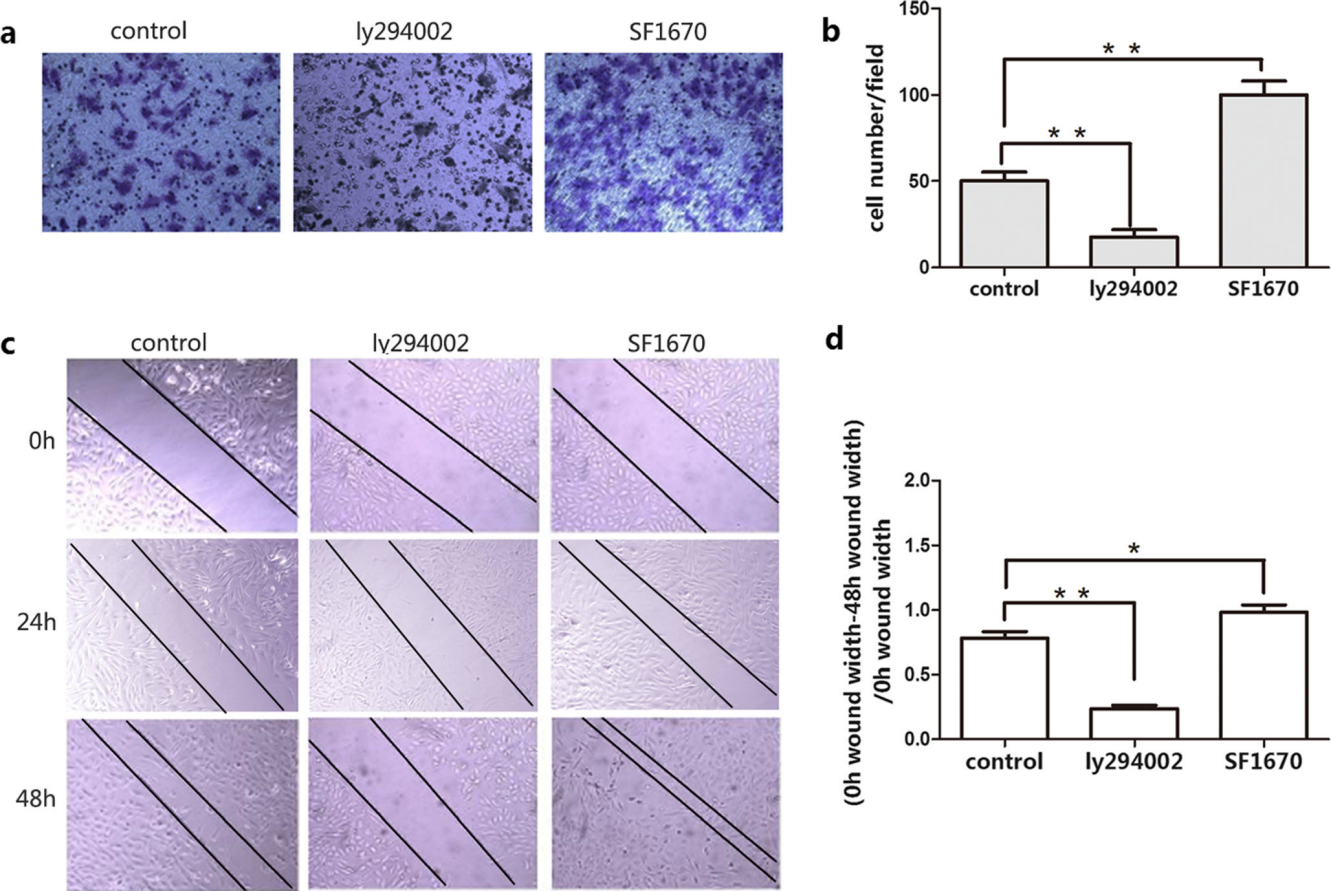
Migration of EcESCs was increased by p-PI3K activation and decreased by p-PI3K inhibition. **a**, **b** Cell migration was detected by transwell assay (200 × scale, *n* = 8). **c**, **d** Cell migration was detected by wound scratch assay (100 × scale, *n* = 8). Results are presented as the mean ± SEM (*p < 0.05, **p < 0.01)

The influence of p-PI3K inhibition and activation on cellular migration of EcESCs was also assessed by transwell assay and wound scratch assays. Migration ability of EcESCs was significantly decreased by ly294002 and increased by SF1670 after 24-h and 48-h treatment (*P* < 0.05, Fig. 6a–d).

## Discussion

Endometriosis is a common but complex disease with undetermined etiology and pathogenesis. OPN plays a crucial role in progression and metastasis in cancer through cell migration, invasion, anti-apoptosis, angiogenesis, and abnormal activated immunocytes. Overexpression of OPN has been reported to promote cell migration in many cancers, such as human nasopharyngeal carcinoma, breast cancer, colorectal cancer, human lung adenocarcinoma, and human endometrial carcinoma [42–45]. Recently, it was also reported that OPN may correlate with the migration of endometrial cells in patients with endometriosis, while the underlying molecular mechanism remained undetermined. It was reported that multifarious biological function of OPN may achieve through the activation of PI3K/Akt pathway by upregulating uPA [25, 26]. Recent studies have showed that ectopic lesion occurs and develops accompanied by cell migration and invasion, and its key regulators have not been illustrated. This prompted us to detect the role of OPN, p-PI3K, and uPA in endometriosis.

Phosphorylation of PI3K has been reported to mediate cellular migration in many malignant tumors, such as human osteosarcoma [46], breast cancer [47], melanoma [48], and hepatoma [49]. uPA, part of the plasminogen-activating system, is known to participate in degradation of extracellular matrix and modulation of cell adhesion and migration [50]. It was reported that migration and invasion of human nasopharyngeal carcinoma cells were suppressed by inhibiting uPA through the modulation of the PI3K/Akt signaling pathway [51]. CD40-related uPA induced human multiple myeloma cell migration through the PI3Ksignalling pathway [52]. We demonstrated that p-PI3K and uPA were upregulated in ESCs from patients with endometriosis and knockdown of OPN decreased the expression of p-PI3K and uPA in EcESCs. Our finding is consistent with several previous studies in which OPN was reported to be related to the PI3K pathway and uPA. It was observed that uPA and p-PI3K were regulated by OPN in gastric cancer [27]. The PI3K signaling pathway was reported to be involved in the progression of ovarian cancer mediated by OPN [40]. Additionally, OPN contributes to the migration of lung cancer cells through combining with alpha v beta 3 integrin or adhensive glycoprotein receptor CD44 by activating the PI3K pathway [53]. OPN and uPA were functionally relevant since OPN-induced enhancement of invasiveness in human mammary epithelial cells was uPA dependent [25, 53, 54]. Moreover, knockdown of OPN significantly reduced the level of uPA and suppressed non-small lung cancer cell invasion and metastasis [55].

To further demonstrate the OPN/p-PI3K/uPA pathway performances vital function in endometriosis, we used 50 μM of ly294002 and 4 μM of SF1670 after 12-h and 24-h treatment to inhibit and activate the p-PI3K pathway respectively. We found that inhibition and activation of the p-PI3K pathway significantly upregulate and downregulate uPA expression, while no apparent change in OPN expression was observed. This result suggests that p-PI3K acts upstream of uPA and downstream of OPN, which means that OPN induces the expression of uPA through the PI3K pathway in endometriosis. In addition, OPN may induce kinds of transcription factors including NF-κB and AP-1 combine with DNA, enhance uPA and MMP synthesis and celluar matrix degradation, inhibit cell apoptosis, and promote proliferation and invasion of ectopic endometrial cell.

Cytoskeleton reorganization was observed simultaneously with decreased p-PI3K and uPA expressions after knockdown of OPN in EcESCs. Specially, significantly decreased fluorescence intensity of α-tubulin rather than F-actin was observed in siRNA-OPN-treated EcESCs. Cells treated with siRNA-OPN changed to a spindle with more thin edges and corners, implying cells have weak migration ability. These results are in accordance with previous reports as tubulin decreased with OPN in response to mechanical strain in osteoblast while neither the level of actin nor that of the intermediate filament protein vimentin changed [41]. We concluded that OPN/p-PI3K/uPA may regulate cytoskeleton reorganization of ectopic ESCs, especially α-tubulin alterations in cell morphology and motility, promoting migration and invasion of ectopic ESCs in endometriosis.

Excessive proliferation of ESCs is also known to contribute to the pathogenesis of endometriosis. OPN has been reported to regulate cell proliferation in ovarian cancer cells [56], nasopharyngeal carcinoma cells [45], and human lung adenocarcinoma cells [44]. In our study, cellular proliferation of EcESCs was not influenced after siRNA-OPN treatment at all set time points. This result may suggest that the major effects of OPN on ESCs might be regulation of cell migration rather than proliferation, while other study reported proliferation in endometrial epithelial cells involved in the pathogenesis of endometriosis [31]. The inconsistent results may due to the different OPN expression in epithelial cells and stromal cells. We performed the correlation analysis, showing serum OPN positive correlated with endometriotic cyst tissue, indicating that serum OPN might be a potential prediction of endometriosis progression.

Actually, there are several limitations in our study. Firstly, the control participants are not disease free due to ethical permission. We set inclusion criteria scrupulously to minimize the impact of other diseases and baseline characteristic between the two groups, such as age, body mass index, parity, and hormone level. Secondly, our study only incorporated endometria specimen of stage III/IV. In future investigation, early stage endometriosis (I/II) would be included to explore the role of OPN in endometriosis progression. Finally, animal experiment would be performed to further identify the role of OPN in endometriosis in vivo.

In summary, we demonstrated that OPN was upregulated in ESCs from participants with endometriosis. OPN played a role in cytoskeleton reorganization through α-tubulin rather than F-actin and regulate cellular migration of ESCs by modulating uPA expression through the PI3K signaling pathway. Despite the limitations, this study does provide us with a better understanding of the pathogenesis of endometriosis and imply potential targets for endometriosis.

## Abbreviations

PI3K: Phosphatidylinositol 3 kinase
uPA: Urokinase plasminogen activator

## References

[1] Sampson JA (1927). Metastatic or embolic endometriosis, due to the menstrual dissemination of endometrial tissue into the venous circulation. Am J Pathol.

[2] Halme J, Hammond MG, Hulka JF, Raj SG, Talbert LM (1984). Retrograde menstruation in healthy women and in patients with endometriosis. Obstet Gynecol.

[3] Cramer DW, Missmer SA (2002). The epidemiology of endometriosis. Ann N Y Acad Sci.

[4] D’Amico F, Skarmoutsou E, Quaderno G, Malaponte G, La Corte C, Scibilia G (2013). Expression and localisation of osteopontin and prominin-1 (CD133) in patients with endometriosis. Int J Mol Med.

[5] Hapangama DK, Turner MA, Drury JA, Martin-Ruiz C, Von Zglinicki T, Farquharson RG (2008). Endometrial telomerase shows specific expression patterns in different types of reproductive failure. Reprod BioMed Online.

[6] Hapangama DK, Turner MA, Drury JA, Quenby S, Saretzki G, Martin-Ruiz C, von Zglinicki T (2008). Endometriosis is associated with aberrant endometrial expression of telomerase and increased telomere length. Hum Reprod.

[7] Deevey S (2005). Endometriosis: internet resources. Med Ref Serv Q.

[8] Bulun SE (2009). Endometriosis. N Engl J Med.

[9] Bodner K, Zauner M, Bodner-Adler B, Spangler B, Grunberger W, Wierrani F (2003). Parenchymatous pulmonary endometriosis - metastases of a low-grade endometrial stromal sarcoma?. Med Hypotheses.

[10] Kwak TK, Sohn EJ, Kim S, Won G, Choi JU, Jeong K, Jeong M, Kwon OS, Kim SH (2014). Inhibitory effect of ethanol extract of Ocimum sanctum on osteopontin mediated metastasis of NCI-H460 non-small cell lung cancer cells. BMC Complement Altern Med.

[11] Reinholt FP, Hultenby K, Oldberg A, Heinegard D (1990). Osteopontin--a possible anchor of osteoclasts to bone. Proc Natl Acad Sci U S A.

[12] Denhardt DT, Guo X (1993). Osteopontin: a protein with diverse functions. FASEB J.

[13] Craig AM, Denhardt DT (1991). The murine gene encoding secreted phosphoprotein 1 (osteopontin): promoter structure, activity, and induction in vivo by estrogen and progesterone. Gene..

[14] O’Regan A, Berman JS (2000). Osteopontin: a key cytokine in cell-mediated and granulomatous inflammation. Int J Exp Pathol.

[15] Weber GF (2001). The metastasis gene osteopontin: a candidate target for cancer therapy. Biochim Biophys Acta.

[16] Standal T, Borset M, Sundan A (2004). Role of osteopontin in adhesion, migration, cell survival and bone remodeling. Exp Oncol.

[17] Rodrigues LR, Teixeira JA, Schmitt FL, Paulsson M, Lindmark-Mansson H (2007). The role of osteopontin in tumor progression and metastasis in breast cancer. Cancer Epidemiol Biomark Prev.

[18] Ostheimer C, Schweyer F, Reese T, Bache M, Vordermark D (2016). The relationship between tumor volume changes and serial plasma osteopontin detection during radical radiotherapy of non-small-cell lung cancer. Oncol Lett.

[19] Ramachandran S, Kwon KY, Shin SJ, Kwon SH, Cha SD, Lee HG, Hong YB, Bae I, Lee GH, Cho CH (2013). Regulatory role of osteopontin in malignant transformation of endometrial cancer. Mol Biol Rep.

[20] Tuck AB, Hota C, Wilson SM, Chambers AF (2003). Osteopontin-induced migration of human mammary epithelial cells involves activation of EGF receptor and multiple signal transduction pathways. Oncogene..

[21] Tuck AB, Elliott BE, Hota C, Tremblay E, Chambers AF (2000). Osteopontin-induced, integrin-dependent migration of human mammary epithelial cells involves activation of the hepatocyte growth factor receptor (Met). J Cell Biochem.

[22] Hayashi C, Rittling S, Hayata T, Amagasa T, Denhardt D, Ezura Y, Nakashima K, Noda M (2007). Serum osteopontin, an enhancer of tumor metastasis to bone, promotes B16 melanoma cell migration. J Cell Biochem.

[23] Slomovitz BM, Coleman RL (2012). The PI3K/AKT/mTOR pathway as a therapeutic target in endometrial cancer. Clin Cancer Res.

[24] Song G, Cai QF, Mao YB, Ming YL, Bao SD, Ouyang GL (2008). Osteopontin promotes ovarian cancer progression and cell survival and increases HIF-1alpha expression through the PI3-K/Akt pathway. Cancer Sci.

[25] Tuck AB, Hota C, Chambers AF (2001). Osteopontin(OPN)-induced increase in human mammary epithelial cell invasiveness is urokinase (uPA)-dependent. Breast Cancer Res Treat.

[26] Chen RX, Xia YH, Xue TC, Ye SL (2011). Osteopontin promotes hepatocellular carcinoma invasion by up-regulating MMP-2 and uPA expression. Mol Biol Rep.

[27] Liu J, Liu Q, Wan Y, Zhao Z, Yu H, Luo H (2014). Osteopontin promotes the progression of gastric cancer through the NF-kappaB pathway regulated by the MAPK and PI3K. Int J Oncol.

[28] Hapangama DK, Raju RS, Valentijn AJ, Barraclough D, Hart A, Turner MA, Platt-Higgins A, Barraclough R, Rudland PS (2012). Aberrant expression of metastasis-inducing proteins in ectopic and matched eutopic endometrium of women with endometriosis: implications for the pathogenesis of endometriosis. Hum Reprod.

[29] Cho S, Ahn YS, Choi YS, Seo SK, Nam A, Kim HY, Kim JH, Park KH, Cho DJ, Lee BS (2009). Endometrial osteopontin mRNA expression and plasma osteopontin levels are increased in patients with endometriosis. Am J Reprod Immunol.

[30] Odagiri K, Konno R, Fujiwara H, Netsu S, Ohwada M, Shibahara H, Suzuki M (2007). Immunohistochemical study of osteopontin and L-selectin in a rat endometriosis model and in human endometriosis. Fertil Steril.

[31] Yang M, Jiang C, Chen H, Nian Y, Bai Z, Ha C (2015). The involvement of osteopontin and matrix metalloproteinase- 9 in the migration of endometrial epithelial cells in patients with endometriosis. Reprod Biol Endocrinol.

[32] Konno R, Fujiwara H, Netsu S, Odagiri K, Shimane M, Nomura H, Suzuki M (2007). Gene expression profiling of the rat endometriosis model. Am J Reprod Immunol.

[33] Yu J, Boicea A, Barrett KL, James CO, Bagchi IC, Bagchi MK, Nezhat C, Sidell N, Taylor RN (2014). Reduced connexin 43 in eutopic endometrium and cultured endometrial stromal cells from subjects with endometriosis. Mol Hum Reprod.

[34] Berbic M, Ng CH, Black K, Markham R, Russell P, Basten A (2013). A novel pilot study of endometrial stromal cells and immune cell populations in sentinel uterine-draining lymph nodes during the menstrual cycle and in endometriosis. Reprod Sci.

[35] Surrey ES, Halme J (1991). Effect of platelet-derived growth factor on endometrial stromal cell proliferation in vitro: a model for endometriosis?. Fertil Steril.

[36] Surrey ES, Halme J (1990). Effect of peritoneal fluid from endometriosis patients on endometrial stromal cell proliferation in vitro. Obstet Gynecol.

[37] Cory G (2011). Scratch-wound assay. Methods Mol Biol.

[38] Arranz-Valsero I, Soriano-Romani L, Garcia-Posadas L, Lopez-Garcia A, Diebold Y (2014). IL-6 as a corneal wound healing mediator in an in vitro scratch assay. Exp Eye Res.

[39] Jonkman JE, Cathcart JA, Xu F, Bartolini ME, Amon JE, Stevens KM (2014). An introduction to the wound healing assay using live-cell microscopy. Cell Adhes Migr.

[40] Kang JA, Zhou Y, Weis TL, Liu H, Ulaszek J, Satgurunathan N, Zhou L, van Besien K, Crispino J, Verma A, Low PS, Wickrema A (2008). Osteopontin regulates actin cytoskeleton and contributes to cell proliferation in primary erythroblasts. J Biol Chem.

[41] Meazzini MC, Toma CD, Schaffer JL, Gray ML, Gerstenfeld LC (1998). Osteoblast cytoskeletal modulation in response to mechanical strain in vitro. J Orthop Res.

[42] Pio GM, Xia Y, Piaseczny MM, Chu JE, Allan AL (2017). Soluble bone-derived osteopontin promotes migration and stem-like behavior of breast cancer cells. PLoS One.

[43] Huang RH, Quan YJ, Chen JH, Wang TF, Xu M, Ye M, Yuan H, Zhang CJ, Liu XJ, Min ZJ (2017). Osteopontin promotes cell migration and invasion, and inhibits apoptosis and autophagy in colorectal cancer by activating the p38 MAPK signaling pathway. Cell Physiol Biochem.

[44] Abdulrahman N, Jaballah M, Poomakkoth N, Riaz S, Abdelaziz S, Issa A, Mraiche F (2016). Inhibition of p90 ribosomal S6 kinase attenuates cell migration and proliferation of the human lung adenocarcinoma through phospho-GSK-3beta and osteopontin. Mol Cell Biochem.

[45] Qin H, Wang R, Wei G, Wang H, Pan G, Hu R, Wei Y, Tang R, Wang J (2018). Overexpression of osteopontin promotes cell proliferation and migration in human nasopharyngeal carcinoma and is associated with poor prognosis. Eur Arch Otorhinolaryngol.

[46] Zhang A, He S, Sun X, Ding L, Bao X, Wang N (2014). Wnt5a promotes migration of human osteosarcoma cells by triggering a phosphatidylinositol-3 kinase/Akt signals. Cancer Cell Int.

[47] Boulay PL, Cotton M, Melancon P, Claing A (2008). ADP-ribosylation factor 1 controls the activation of the phosphatidylinositol 3-kinase pathway to regulate epidermal growth factor-dependent growth and migration of breast cancer cells. J Biol Chem.

[48] Shin DH, Kim OH, Jun HS, Kang MK (2008). Inhibitory effect of capsaicin on B16-F10 melanoma cell migration via the phosphatidylinositol 3-kinase/Akt/Rac1 signal pathway. Exp Mol Med.

[49] Shih WL, Liao MH, Yu FL, Lin PY, Hsu HY, Chiu SJ (2008). AMF/PGI transactivates the MMP-3 gene through the activation of Src-RhoA-phosphatidylinositol 3-kinase signaling to induce hepatoma cell migration. Cancer Lett.

[50] Bruse C, Radu D, Bergqvist A (2004). In situ localization of mRNA for the fibrinolytic factors uPA, PAI-1 and uPAR in endometriotic and endometrial tissue. Mol Hum Reprod.

[51] Ho HY, Ho YC, Hsieh MJ, Yang SF, Chuang CY, Lin CW, Hsin CH (2017). Hispolon suppresses migration and invasion of human nasopharyngeal carcinoma cells by inhibiting the urokinase-plasminogen activator through modulation of the Akt signaling pathway. Environ Toxicol.

[52] Tai YT, Podar K, Mitsiades N, Lin B, Mitsiades C, Gupta D, Akiyama M, Catley L, Hideshima T, Munshi NC, Treon SP, Anderson KC (2003). CD40 induces human multiple myeloma cell migration via phosphatidylinositol 3-kinase/AKT/NF-kappa B signaling. Blood..

[53] Fong YC, Liu SC, Huang CY, Li TM, Hsu SF, Kao ST, Tsai FJ, Chen WC, Chen CY, Tang CH (2009). Osteopontin increases lung cancer cells migration via activation of the alphavbeta3 integrin/FAK/Akt and NF-kappaB-dependent pathway. Lung Cancer.

[54] Vaschetto R, Navalesi P, Clemente N, Boggio E, Valsecchi S, Olivieri C, Soluri MF, Kroumova V, Fonio P, Dinatale C, Borrè S, Fortina G, Umberto D, Della Corte F, Chiocchetti A (2015). Osteopontin induces soluble urokinase-type plasminogen activator receptor production and release. Minerva Anestesiol.

[55] Sun BS, You J, Li Y, Zhang ZF, Wang CL (2013). Osteopontin knockdown suppresses non-small cell lung cancer cell invasion and metastasis. Chin Med J.

[56] Xu C, Li H, Yin M, Yang T, An L, Yang G (2017). Osteopontin is involved in TLR4 pathway contributing to ovarian cancer cell proliferation and metastasis. Oncotarget..

